# Supplementary material for: Bidirectional yet asymmetric causality between urban systems and traffic dynamics in 30 cities worldwide
Source: Nat Commun. 2026 Apr 1;17:4671. doi: 10.1038/s41467-026-71377-0 (PMC13201568; doi:10.1038/s41467-026-71377-0)
Supplement: Supplementary file 1 — Supplementary Information [file 41467_2026_71377_MOESM1_ESM.pdf]

Supplementary Information

Bidirectional yet asymmetric causality between urban systems and traffic dynamics in 30 cities worldwide

Yatao Zhang[a,b,c,*], Ye Hong[b], Song Gao[d], Martin Raubal[b,a]

[a] *Future Resilient Systems, Singapore-ETH Centre, ETH Zurich, Singapore, Singapore*
[b] *Institute of Cartography and Geoinformation, ETH Zurich, Zurich, Switzerland*
[c] *Department of Geography, University College London, London, United Kingdom*
[d] *Geospatial Data Science Lab, Department of Geography, University of Wisconsin-Madison, Madison, WI, USA*

[*] *Corresponding author: yatzhang@ethz.ch*

List of Supplementary Items

Supplementary Figures **3**

Supplementary Fig. 1: Spatio-temporal association between urban systems and traffic dynamics during work days across 30 cities . . . 3

Supplementary Fig. 2: Bidirectional causal patterns between urban systems and traffic dynamics during congestion periods on work days across 30 cities . . . 4

Supplementary Fig. 3: Heatmap of used features in the STWR model across 30 cities by urban system component . . . 5

Supplementary Fig. 4: Average ρ values at the largest library size L with varying E on rest days across 30 cities . . . 6

Supplementary Fig. 5: Average ρ values at the largest library size L with varying E on work days across 30 cities . . . 7

Supplementary Tables **8**

Supplementary Table 1: R^2, adjusted α, and adjusted critical t values quantifying the association between urban systems and traffic dynamics across 30 cities on rest days . . . 8

Supplementary Table 2: R^2, adjusted α (95%), and adjusted critical t (95%) values quantifying the association between urban systems and traffic dynamics across 30 cities on work days . . . 9

Supplementary Table 3: Performance comparison of STWR and GWR in quantifying associations between urban systems and traffic dynamics on rest days . . . 10

Supplementary Table 4: Performance comparison of STWR and GWR in quantifying associations between urban systems and traffic dynamics on work days . . . 11

Supplementary Table 5: Average ρ values with varying library sizes L reflecting the causal influence between urban systems and traffic dynamics on rest days across 30 cities . . . 12

Supplementary Table 6: Average ρ values with varying library sizes L reflecting the causal influence between urban systems and traffic dynamics on work days across 30 cities . . . 13

Supplementary Table 7: Statistical assessment of the causal asymmetry ($\Delta\rho$) between urban systems and traffic dynamics across 30 cities . . . 14

Supplementary Table 8: Spatial and temporal details of the HERE traffic dataset for 30 global cities 15

Supplementary Table 9: Descriptions of features characterizing urban structure, form, and function 17

Supplementary Figures

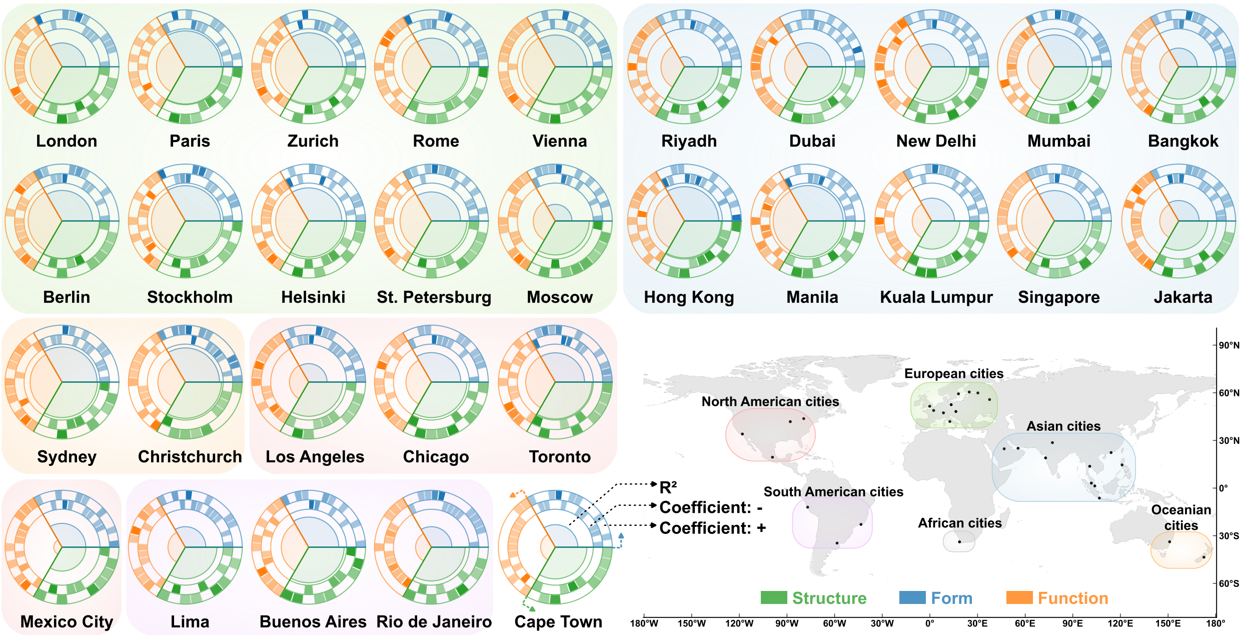

Supplementary Fig. 1: Spatio-temporal association between urban systems and traffic dynamics during work days across 30 cities, with STWR results for structure, form, and function. Each city is visualized as a concentric circle, with the inner circle indicating its R^2 value, the middle ring showing negative STWR coefficients, and the outer ring displaying positive STWR coefficients. Administrative boundaries are derived from the Global Administrative Boundaries (GADM).

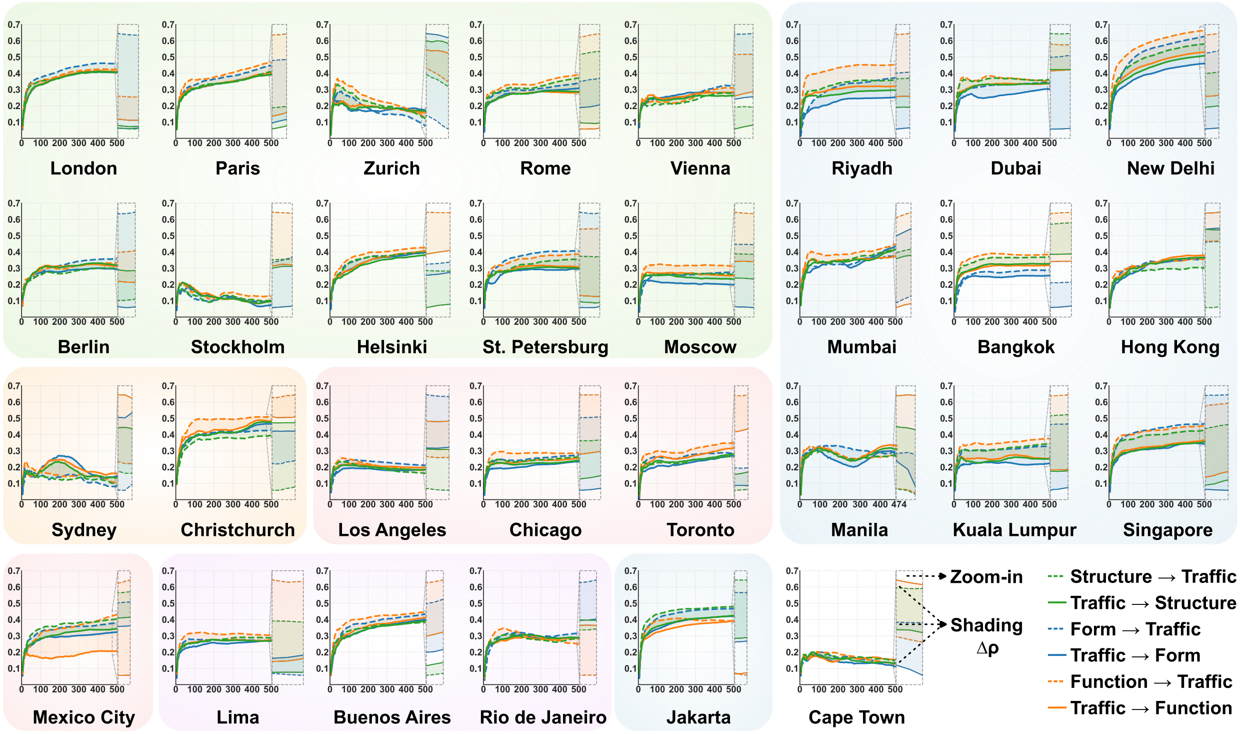

Supplementary Fig. 2: Bidirectional causal patterns between urban systems and traffic dynamics during congestion periods on work days across 30 cities. The L-ρ plots illustrate STCCM results for each city, with structure, form, and function. Increasing ρ values with larger library sizes (L) indicate the presence of spatial causality.

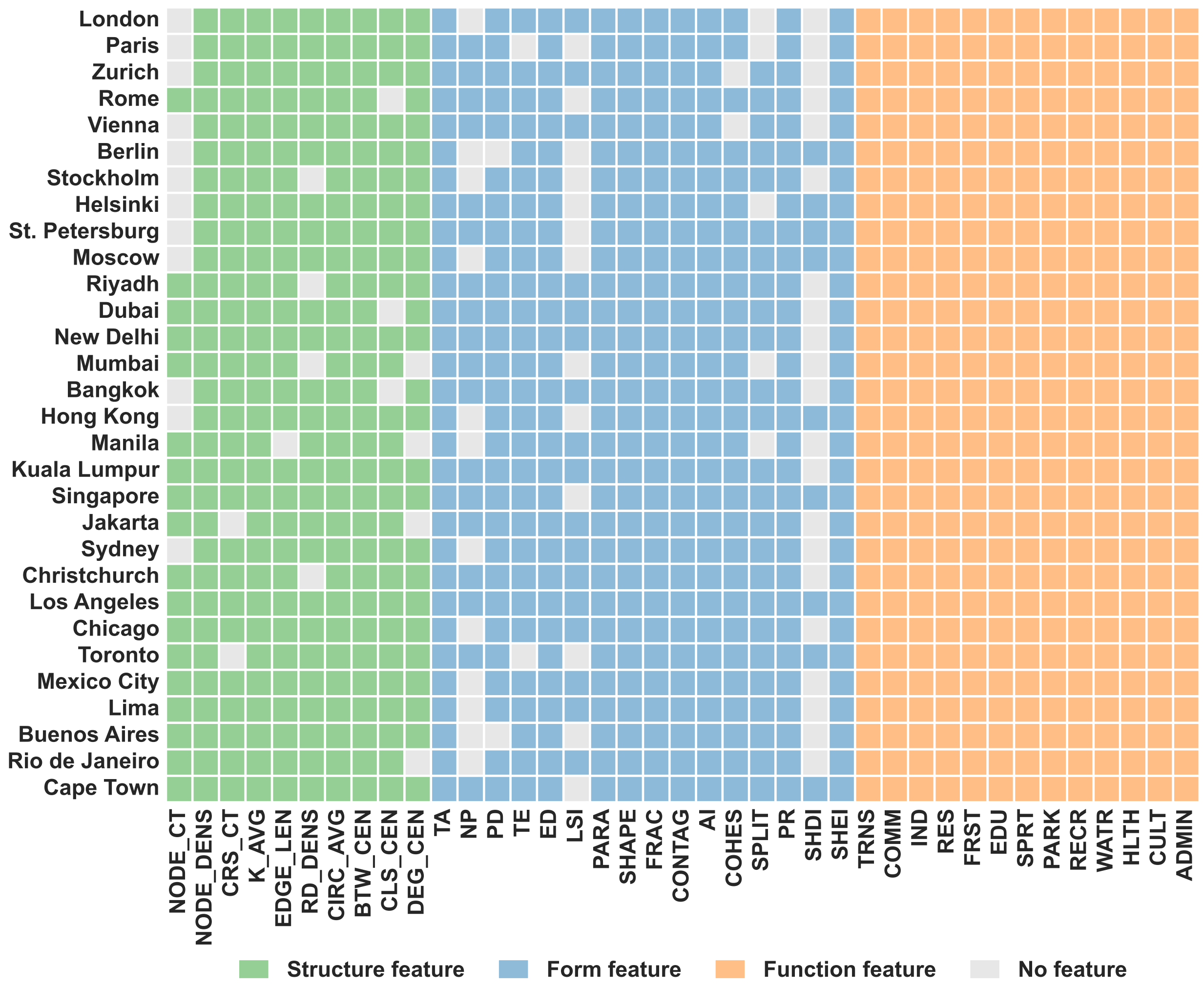

Supplementary Fig. 3: Heatmap of used features in the STWR model across 30 cities by urban system component. To mitigate multicollinearity, we apply variance inflation factor (VIF) screening and iteratively remove features with VIF > 10 before fitting the STWR model. Each row corresponds to a city, and columns list features for urban structure, form, and function, respectively. Definitions of all feature abbreviations are provided in Supplementary Table 9. Colored cells indicate features retained in the STWR estimation, whereas grey cells mark features excluded.

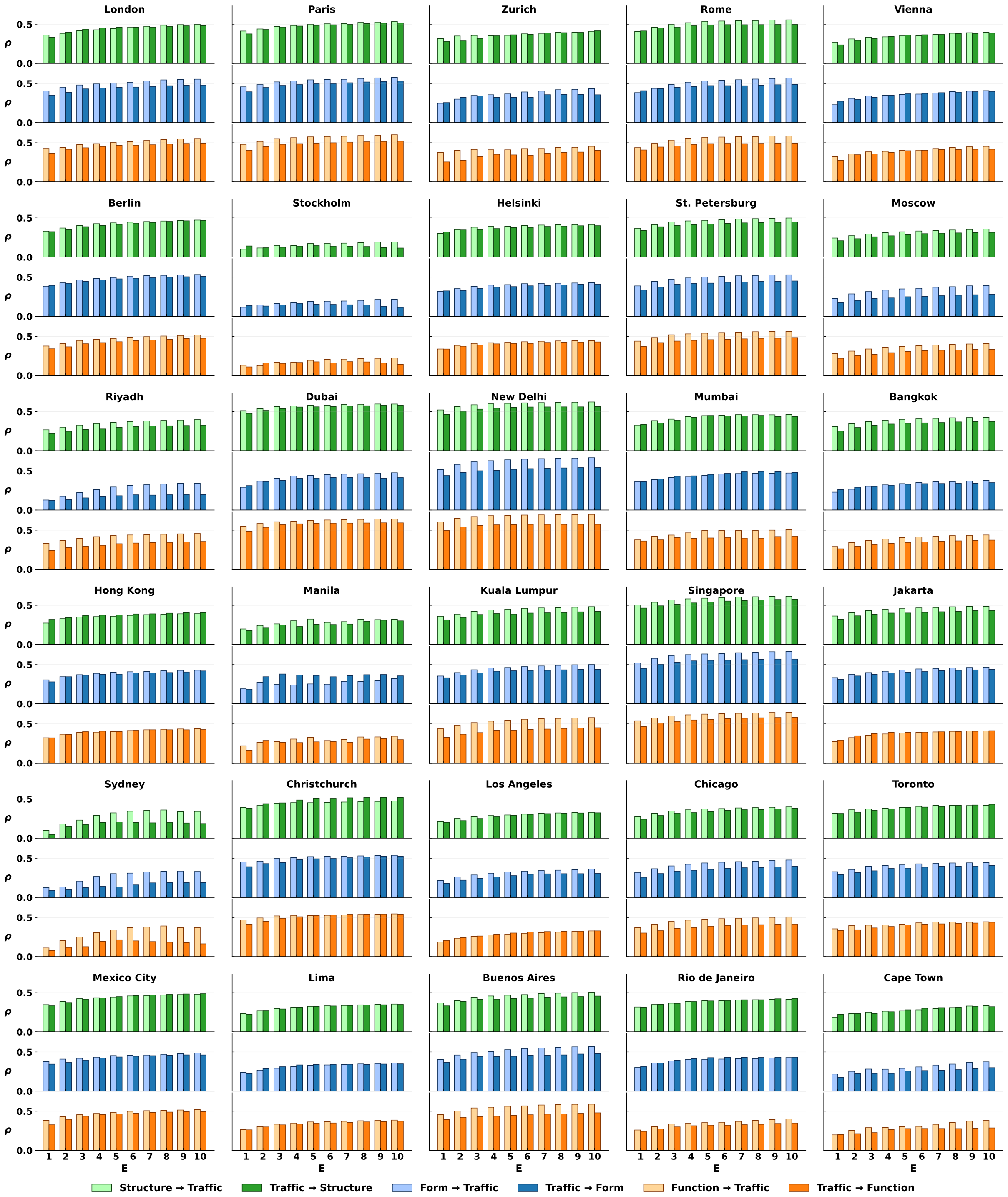

Supplementary Fig. 4: Average ρ values at the largest library size L with varying E on rest days across 30 cities (n = number of BVCells, varying by city). E denotes the embedding dimension for state space reconstruction. We evaluated STCCM performance with E ranging from 1 to 10. Generally, increasing E leads to higher ρ values, but the performance stabilizes around $E = 7$ across most cities. For both robustness and computational efficiency, we therefore select $E = 7$ as the embedding dimension for reconstructing the state space in causal inference.

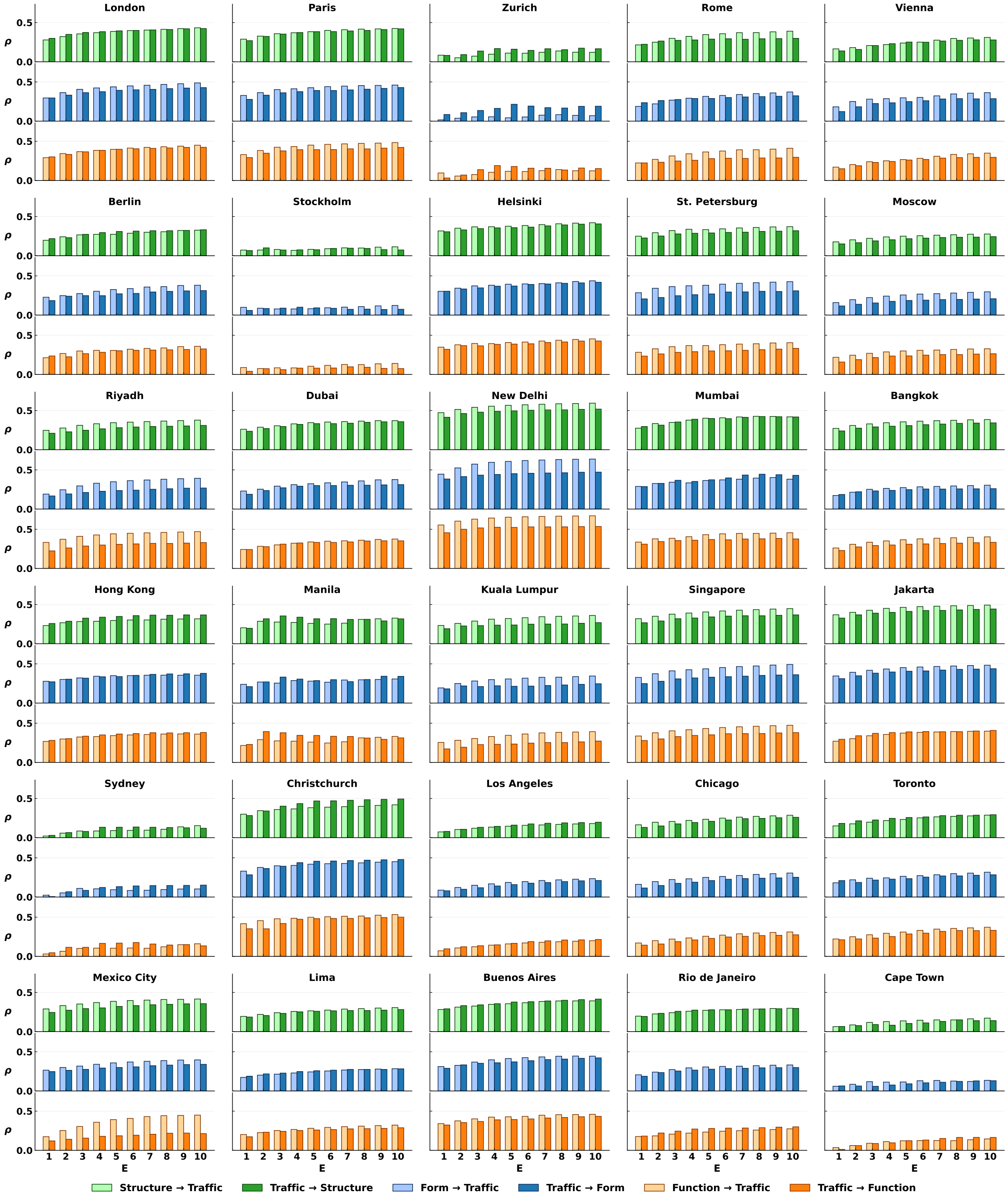

Supplementary Fig. 5: Average ρ values at the largest library size L with varying E on work days across 30 cities (n = number of BVCells, varying by city). E denotes the embedding dimension for state space reconstruction. The trend is similar to that observed on rest days (see Supplementary Fig. 4). For consistency, we also select $E = 7$ as the embedding dimension for reconstructing the state space in causal inference.

Supplementary Tables

Supplementary Table 1: R^2, adjusted α, and adjusted critical t values quantifying the association between urban systems and traffic dynamics, derived from the STWR model of urban structure, form, and function across 30 cities on rest days. All inferential thresholds are reported at the 95% confidence level, with adjusted α accounting for multiple comparisons and the associated critical t denoting the decision boundary for local parameter significance. Since the STWR model estimates location- and time-specific coefficients, it involves many simultaneous local tests. Therefore, we report these adjusted measures to ensure robust and comparable significance assessments.

City	Urban structure			Urban form			Urban function		
	R^2	Adj. α (95%)	Adj. t (95%)	R^2	Adj. α (95%)	Adj. t (95%)	R^2	Adj. α (95%)	Adj. t (95%)
London	0.98	0.0004	3.55	0.77	0.0014	3.20	0.80	0.0012	3.23
Paris	0.97	0.0006	3.41	0.93	0.0010	3.29	0.78	0.0019	3.11
Zurich	0.97	0.0015	3.18	0.96	0.0024	3.04	0.87	0.0028	2.99
Rome	0.97	0.0004	3.53	0.90	0.0008	3.35	0.89	0.0008	3.37
Vienna	0.98	0.0008	3.36	0.92	0.0012	3.24	0.82	0.0016	3.15
Berlin	0.99	0.0006	3.44	0.76	0.0091	2.61	0.77	0.0019	3.10
Stockholm	0.98	0.0008	3.36	0.94	0.0015	3.17	0.91	0.0016	3.15
Helsinki	0.97	0.0004	3.56	0.92	0.0007	3.41	0.88	0.0007	3.39
St. Petersburg	0.89	0.0003	3.65	0.78	0.0008	3.36	0.79	0.0007	3.38
Moscow	0.93	0.0001	3.83	0.52	0.0005	3.49	0.61	0.0004	3.57
Riyadh	0.91	0.0002	3.69	0.26	0.0026	3.01	0.70	0.0006	3.43
Dubai	0.96	0.0003	3.60	0.50	0.0022	3.06	0.66	0.0017	3.14
New Delhi	0.92	0.0002	3.67	0.66	0.0008	3.37	0.68	0.0008	3.36
Mumbai	0.95	0.0009	3.34	0.90	0.0019	3.12	0.79	0.0023	3.05
Bangkok	0.87	0.0002	3.77	0.50	0.0008	3.37	0.49	0.0007	3.39
Hong Kong	0.97	0.0004	3.55	0.87	0.0010	3.29	0.79	0.0011	3.25
Manila	0.95	0.0019	3.11	0.94	0.0032	2.96	0.90	0.0036	2.92
Kuala Lumpur	0.91	0.0006	3.45	0.80	0.0011	3.28	0.68	0.0017	3.14
Singapore	0.95	0.0003	3.58	0.75	0.0010	3.29	0.89	0.0006	3.43
Jakarta	0.84	0.0002	3.69	0.68	0.0006	3.45	0.52	0.0011	3.27
Sydney	0.97	0.0019	3.11	0.95	0.0033	2.95	0.87	0.0055	2.78
Christchurch	0.96	0.0008	3.35	0.82	0.0027	3.00	0.81	0.0025	3.03
Los Angeles	0.92	0.0001	3.85	0.50	0.0007	3.41	0.52	0.0007	3.38
Chicago	0.96	0.0002	3.72	0.72	0.0009	3.33	0.72	0.0009	3.33
Toronto	0.98	0.0006	3.45	0.91	0.0012	3.25	0.87	0.0011	3.26
Mexico City	0.94	0.0005	3.47	0.91	0.0009	3.33	0.75	0.0011	3.26
Lima	0.87	0.0002	3.71	0.58	0.0006	3.44	0.59	0.0004	3.53
Buenos Aires	0.95	0.0006	3.43	0.76	0.0012	3.24	0.68	0.0016	3.15
Rio de Janeiro	0.92	0.0002	3.67	0.56	0.0009	3.31	0.35	0.0016	3.15
Cape Town	0.91	0.0006	3.41	0.65	0.0019	3.11	0.47	0.0029	2.98

Supplementary Table 2: R^2, adjusted α (95%), and adjusted critical t (95%) values quantifying the association between urban systems and traffic dynamics, derived from the STWR model of urban structure, form, and function across 30 cities on work days. We apply the same performance measures and significance tests reported in Supplementary Table 1.

City	Urban structure			Urban form			Urban function		
	R^2	Adj. α (95%)	Adj. t (95%)	R^2	Adj. α (95%)	Adj. t (95%)	R^2	Adj. α (95%)	Adj. t (95%)
London	0.98	0.0004	3.55	0.63	0.0011	3.27	0.84	0.0007	3.41
Paris	0.98	0.0006	3.41	0.95	0.0010	3.30	0.76	0.0019	3.11
Zurich	0.97	0.0015	3.18	0.90	0.0025	3.03	0.88	0.0027	3.00
Rome	0.96	0.0004	3.52	0.75	0.0009	3.33	0.77	0.0008	3.35
Vienna	0.98	0.0008	3.36	0.84	0.0015	3.18	0.87	0.0015	3.18
Berlin	0.98	0.0006	3.44	0.81	0.0017	3.15	0.89	0.0010	3.29
Stockholm	0.96	0.0008	3.36	0.93	0.0015	3.18	0.89	0.0017	3.14
Helsinki	0.95	0.0004	3.55	0.87	0.0007	3.40	0.87	0.0007	3.39
St. Petersburg	0.94	0.0002	3.67	0.77	0.0006	3.44	0.83	0.0005	3.49
Moscow	0.89	0.0001	3.85	0.44	0.0006	3.43	0.58	0.0004	3.56
Riyadh	0.90	0.0002	3.69	0.27	0.0022	3.06	0.62	0.0008	3.34
Dubai	0.92	0.0003	3.60	0.63	0.0010	3.30	0.61	0.0012	3.23
New Delhi	0.92	0.0002	3.69	0.60	0.0010	3.29	0.69	0.0007	3.40
Mumbai	0.89	0.0009	3.33	0.84	0.0018	3.12	0.70	0.0023	3.05
Bangkok	0.85	0.0002	3.77	0.49	0.0010	3.28	0.50	0.0007	3.38
Hong Kong	0.97	0.0004	3.55	0.90	0.0007	3.38	0.81	0.0011	3.25
Manila	0.94	0.0019	3.11	0.93	0.0031	2.97	0.91	0.0037	2.91
Kuala Lumpur	0.74	0.0007	3.38	0.60	0.0014	3.19	0.51	0.0019	3.10
Singapore	0.91	0.0003	3.58	0.80	0.0008	3.36	0.86	0.0006	3.43
Jakarta	0.77	0.0003	3.64	0.54	0.0007	3.40	0.50	0.0011	3.27
Sydney	0.93	0.0020	3.10	0.93	0.0033	2.94	0.84	0.0040	2.89
Christchurch	0.96	0.0008	3.35	0.90	0.0016	3.16	0.72	0.0026	3.01
Los Angeles	0.90	0.0001	3.83	0.49	0.0007	3.40	0.51	0.0007	3.41
Chicago	0.94	0.0002	3.70	0.78	0.0005	3.51	0.67	0.0008	3.36
Toronto	0.97	0.0006	3.45	0.77	0.0015	3.17	0.78	0.0019	3.10
Mexico City	0.63	0.0008	3.36	0.63	0.0011	3.27	0.22	0.0048	2.82
Lima	0.83	0.0002	3.71	0.54	0.0006	3.41	0.46	0.0008	3.36
Buenos Aires	0.94	0.0006	3.42	0.79	0.0010	3.29	0.72	0.0014	3.20
Rio de Janeiro	0.89	0.0002	3.67	0.54	0.0011	3.28	0.42	0.0013	3.23
Cape Town	0.83	0.0007	3.39	0.59	0.0018	3.13	0.39	0.0041	2.87

Supplementary Table 3: Performance comparison of STWR and GWR in quantifying associations between urban systems and traffic dynamics on rest days. The GWR model was estimated for a one-hour time slice (12:00–13:00), with the optimal bandwidth selected via cross-validation. Across all three urban system components, the STWR model consistently outperforms the GWR model in 30 cities, yielding higher R^2 and lower AICc. Note that AICc is a small-sample adjustment of AIC, where lower values indicate models with better expected out-of-sample performance. "-" indicates the estimate is suppressed due to an unstable local fit (e.g., near-zero local variance or insufficient effective neighbors).

City	Urban structure				Urban form				Urban function			
	STWR		GWR		STWR		GWR		STWR		GWR	
	R^2	AICc	R^2	AICc	R^2	AICc	R^2	AICc	R^2	AICc	R^2	AICc
London	0.98	-530.70	0.34	8727.98	0.77	4105.69	0.23	8965.67	0.80	3876.75	0.35	8714.95
Paris	0.97	1502.02	0.51	5000.32	0.93	2759.87	0.20	5369.36	0.78	3800.91	0.43	4961.03
Zurich	0.97	-325.19	0.50	1310.37	0.96	-143.06	0.42	1418.32	0.87	425.54	0.39	1377.09
Rome	0.97	-58.64	0.66	5991.36	0.90	2040.56	0.48	5995.70	0.89	2391.67	0.42	6025.58
Vienna	0.98	-846.17	0.37	3111.24	0.92	1185.44	0.22	3309.13	0.82	1707.15	0.35	3160.27
Berlin	0.99	-3.08	0.30	5539.21	0.76	3267.22	0.21	5599.55	0.77	3808.85	0.29	5554.87
Stockholm	0.98	-786.91	0.35	2856.83	0.94	91.45	0.24	2998.20	0.91	555.08	0.26	2987.82
Helsinki	0.97	-3628.16	0.57	3383.57	0.92	-1389.12	0.49	3773.75	0.88	-568.75	0.40	3966.94
St. Petersburg	0.89	6136.14	0.44	10980.48	0.78	6760.28	0.24	11426.26	0.79	6318.37	0.33	11132.70
Moscow	0.93	4014.38	0.37	19959.63	0.52	14899.34	0.06	20818.96	0.61	13958.04	0.24	20161.90
Riyadh	0.91	6159.88	0.56	11910.30	0.26	10048.02	0.21	10581.66	0.70	9690.86	0.19	13078.94
Dubai	0.96	2052.05	0.48	7448.76	0.50	6922.89	0.26	7798.54	0.66	6368.96	0.25	8438.73
New Delhi	0.92	12505.57	0.57	18434.97	0.66	16022.14	0.36	18133.16	0.68	16283.44	0.32	19577.64
Mumbai	0.95	1974.23	0.50	3779.03	0.90	2161.91	0.16	3262.88	0.79	2950.32	0.11	3929.44
Bangkok	0.87	5443.19	0.60	19969.05	0.50	8259.07	0.23	14508.71	0.49	11128.53	0.12	22654.59
Hong Kong	0.97	-4614.35	0.61	3399.63	0.87	-2189.06	0.12	3964.80	0.79	-1199.71	0.38	3462.58
Manila	0.95	-132.44	0.18	1141.53	0.94	-94.62	0.12	1148.14	0.90	68.26	0.16	1159.07
Kuala Lumpur	0.91	2237.95	0.58	5138.66	0.80	3234.78	0.18	5333.17	0.68	3325.86	-	12305.30
Singapore	0.95	350.83	0.68	6545.35	0.75	3361.08	0.22	7708.88	0.89	1522.40	0.46	7075.73
Jakarta	0.84	4897.83	0.40	13832.49	0.68	6802.54	0.16	14004.19	0.52	7237.28	0.17	14481.70
Sydney	0.97	48.51	0.53	901.63	0.95	341.08	0.20	970.88	0.87	459.34	0.33	955.32
Christchurch	0.96	-1180.33	0.52	1691.43	0.82	-203.75	0.40	1806.18	0.81	-148.19	0.43	1621.76
Los Angeles	0.92	1482.66	0.62	19478.83	0.50	10543.90	0.33	19963.57	0.52	9975.67	0.37	21613.25
Chicago	0.96	-1446.72	0.63	12331.50	0.72	5028.27	0.40	14064.61	0.72	5113.78	0.55	13092.16
Toronto	0.98	-115.36	0.61	4855.84	0.91	2208.60	0.42	5033.16	0.87	2845.99	0.49	5040.74
Mexico City	0.94	2713.77	0.35	6918.89	0.91	3035.38	0.35	6472.36	0.75	4715.65	0.30	7048.92
Lima	0.87	6910.66	0.29	14946.80	0.58	9370.32	0.23	15054.19	0.59	10403.51	0.25	15053.89
Buenos Aires	0.95	1275.11	0.35	4584.62	0.76	3350.52	0.20	4844.73	0.68	3570.99	0.23	4820.14
Rio de Janeiro	0.92	3055.64	0.54	11376.52	0.56	6367.22	0.29	10715.88	0.35	8019.65	0.26	12514.62
Cape Town	0.91	-589.33	0.54	3887.97	0.65	530.89	0.29	4047.26	0.47	781.23	-	7905.57

Supplementary Table 4: Performance comparison of STWR and GWR in quantifying associations between urban systems and traffic dynamics on work days. The GWR model was estimated for a one-hour time slice (08:00-09:00), with the optimal bandwidth selected via cross-validation. The performance comparison is similar to that observed on rest days (see Supplementary Table 3)."-" indicates the estimate is suppressed due to an unstable local fit (e.g., near-zero local variance or insufficient effective neighbors).

City	Urban structure				Urban form				Urban function			
	STWR		GWR		STWR		GWR		STWR		GWR	
	R^2	AICc	R^2	AICc	R^2	AICc	R^2	AICc	R^2	AICc	R^2	AICc
London	0.98	-589.63	0.35	9310.86	0.63	5292.42	0.41	9311.53	0.84	4172.23	0.41	9265.62
Paris	0.98	984.61	0.38	5281.23	0.95	2302.67	0.10	5568.17	0.76	3858.94	0.47	5241.43
Zurich	0.97	-52.77	0.40	1820.34	0.90	644.44	0.30	1919.52	0.88	615.88	0.30	1864.02
Rome	0.96	682.53	0.46	7904.19	0.75	3906.64	0.29	8127.96	0.77	3947.77	0.31	8044.35
Vienna	0.98	-503.60	0.56	3995.14	0.84	1814.22	0.20	4015.10	0.87	1368.69	0.32	3897.33
Berlin	0.98	551.26	0.28	5759.20	0.81	3595.72	0.20	5816.98	0.89	3386.65	0.28	5775.82
Stockholm	0.96	130.04	0.36	2988.83	0.93	366.74	0.29	3089.57	0.89	683.26	0.26	3095.60
Helsinki	0.95	-1685.19	0.69	4576.52	0.87	263.68	0.52	4969.16	0.87	-69.34	0.46	5081.53
St. Petersburg	0.94	4505.18	0.48	13566.56	0.77	8289.34	0.30	13883.42	0.83	7213.09	0.34	13917.91
Moscow	0.89	10074.37	0.37	24710.22	0.44	16460.06	0.03	25639.64	0.58	15513.56	0.23	24881.72
Riyadh	0.90	7186.40	0.53	11763.83	0.27	10226.14	0.25	10278.21	0.62	10319.03	0.18	13034.30
Dubai	0.92	3897.74	0.44	9568.49	0.63	6503.65	0.37	9141.09	0.61	6600.24	0.29	10072.78
New Delhi	0.92	12830.08	0.54	17443.44	0.60	15738.90	0.31	17176.50	0.69	16025.48	0.27	18544.03
Mumbai	0.89	2619.19	0.41	3371.83	0.84	2361.28	0.07	2961.05	0.70	3041.31	0.12	3531.76
Bangkok	0.85	7473.90	0.56	23803.58	0.49	8174.76	0.29	16503.97	0.50	11727.45	0.11	26636.50
Hong Kong	0.97	-3989.65	0.57	5022.55	0.90	-1364.98	0.18	5600.79	0.81	-878.80	0.36	5344.91
Manila	0.94	248.08	0.21	1256.13	0.93	330.68	0.22	1256.92	0.91	324.78	0.23	1259.62
Kuala Lumpur	0.74	2493.24	0.55	6310.14	0.60	2788.41	0.11	6462.72	0.51	2790.07	-	17798.06
Singapore	0.91	1324.83	0.53	8041.30	0.80	2389.39	0.14	8394.50	0.86	1646.17	0.30	8383.56
Jakarta	0.77	5891.28	0.42	15453.76	0.54	8009.47	0.18	15721.04	0.50	7419.36	0.17	16433.78
Sydney	0.93	185.50	0.30	1079.10	0.93	220.57	0.15	1104.85	0.84	429.88	0.25	1101.50
Christchurch	0.96	-808.30	0.55	2254.14	0.90	-218.34	0.47	2408.90	0.72	446.74	0.46	2307.94
Los Angeles	0.90	2746.60	0.54	24285.61	0.49	10589.29	0.31	23502.20	0.51	10632.89	0.36	25826.00
Chicago	0.94	-488.82	0.62	14138.91	0.78	4736.81	0.40	15673.46	0.67	5366.30	0.53	14936.24
Toronto	0.97	639.24	0.51	4964.15	0.77	3117.13	0.38	5057.34	0.78	2805.91	0.35	5073.41
Mexico City	0.63	6055.62	0.13	7954.64	0.63	5650.65	0.12	7251.59	0.22	6402.39	0.20	7902.26
Lima	0.83	8296.31	0.33	17388.50	0.54	9954.07	0.24	17597.36	0.46	10357.24	0.23	17896.16
Buenos Aires	0.94	1805.20	0.29	5517.40	0.79	3234.22	0.18	5616.34	0.72	3444.27	0.16	5724.89
Rio de Janeiro	0.89	3524.88	0.42	13554.72	0.54	5515.30	0.26	12353.31	0.42	6893.31	0.29	14107.73
Cape Town	0.83	-358.29	0.54	4583.33	0.59	351.36	0.37	4647.10	0.39	367.58	0.28	4798.89

Supplementary Table 5: Average ρ values with varying library sizes L reflecting the causal influence between urban systems and traffic dynamics on rest days across 30 cities. Results are presented for library sizes of 100 and 500, except for Manila, where the maximum available library size is below 500 and the ρ value from the largest available size is used. Statistical significance was evaluated using a one-sample Student's t-test on the distribution of ρ values across all BVCells at each library size. All statistical tests were two-sided. Exact p-values for all statistical tests are provided in the source data files.

	Urban system → Traffic dynamics						Traffic dynamics → Urban system					
City	Structure		Form		Function		Structure		Form		Function	
	100	500	100	500	100	500	100	500	100	500	100	500
London	0.43***	0.47***	0.47***	0.53***	0.47***	0.53***	0.38***	0.46***	0.38***	0.46***	0.38***	0.47***
Paris	0.35***	0.51***	0.39***	0.55***	0.43***	0.58***	0.39***	0.50***	0.39***	0.51***	0.39***	0.51***
Zurich	0.39***	0.38***	0.39***	0.40***	0.44***	0.43***	0.35***	0.39***	0.34***	0.36***	0.35***	0.37***
Rome	0.40***	0.55***	0.40***	0.55***	0.43***	0.58***	0.36***	0.49***	0.36***	0.47***	0.36***	0.49***
Vienna	0.31***	0.37***	0.31***	0.38***	0.34***	0.43***	0.28***	0.37***	0.28***	0.38***	0.30***	0.41***
Berlin	0.37***	0.45***	0.43***	0.52***	0.42***	0.50***	0.39***	0.44***	0.42***	0.49***	0.39***	0.46***
Stockholm	0.17***	0.18***	0.15***	0.20***	0.19***	0.21***	0.17***	0.14***	0.16***	0.15***	0.18***	0.18***
Helsinki	0.33***	0.41***	0.33***	0.42***	0.35***	0.44***	0.30***	0.39***	0.30***	0.39***	0.30***	0.42***
St. Petersburg	0.36***	0.48***	0.40***	0.52***	0.42***	0.55***	0.35***	0.44***	0.35***	0.44***	0.37***	0.47***
Moscow	0.31***	0.34***	0.33***	0.37***	0.37***	0.39***	0.28***	0.31***	0.24***	0.26***	0.29***	0.32***
Riyadh	0.33***	0.38***	0.20***	0.32***	0.40***	0.44***	0.27***	0.32***	0.16***	0.19***	0.30***	0.34***
Dubai	0.51***	0.59***	0.34***	0.46***	0.53***	0.63***	0.48***	0.57***	0.32***	0.41***	0.49***	0.59***
New Delhi	0.47***	0.61***	0.50***	0.66***	0.56***	0.69***	0.41***	0.56***	0.39***	0.53***	0.42***	0.57***
Mumbai	0.39***	0.46***	0.36***	0.47***	0.45***	0.49***	0.36***	0.45***	0.37***	0.49***	0.38***	0.40***
Bangkok	0.37***	0.41***	0.31***	0.36***	0.40***	0.42***	0.32***	0.36***	0.27***	0.34***	0.31***	0.36***
Hong Kong	0.29***	0.38***	0.32***	0.41***	0.33***	0.42***	0.29***	0.39***	0.28***	0.39***	0.32***	0.42***
Manila	0.27***	0.29***	0.30***	0.29***	0.30***	0.30***	0.30***	0.26***	0.32***	0.36***	0.32***	0.26***
Kuala Lumpur	0.37***	0.47***	0.37***	0.48***	0.47***	0.57***	0.34***	0.40***	0.34***	0.43***	0.37***	0.43***
Singapore	0.50***	0.60***	0.54***	0.65***	0.53***	0.63***	0.45***	0.56***	0.45***	0.56***	0.46***	0.57***
Jakarta	0.41***	0.47***	0.40***	0.45***	0.37***	0.40***	0.36***	0.42***	0.36***	0.42***	0.32***	0.40***
Sydney	0.25***	0.35***	0.23***	0.32***	0.29***	0.38***	0.25***	0.19***	0.27***	0.19***	0.25***	0.19***
Christchurch	0.38***	0.46***	0.44***	0.53***	0.45***	0.53***	0.42***	0.51***	0.43***	0.51***	0.45***	0.54***
Los Angeles	0.31***	0.32***	0.32***	0.34***	0.33***	0.31***	0.30***	0.31***	0.27***	0.30***	0.30***	0.32***
Chicago	0.32***	0.39***	0.37***	0.46***	0.41***	0.49***	0.29***	0.36***	0.27***	0.38***	0.31***	0.40***
Toronto	0.32***	0.42***	0.34***	0.44***	0.36***	0.44***	0.31***	0.40***	0.30***	0.40***	0.34***	0.42***
Mexico City	0.38***	0.46***	0.40***	0.46***	0.43***	0.51***	0.37***	0.47***	0.35***	0.45***	0.39***	0.48***
Lima	0.28***	0.34***	0.31***	0.34***	0.33***	0.37***	0.26***	0.34***	0.26***	0.34***	0.29***	0.36***
Buenos Aires	0.39***	0.49***	0.43***	0.55***	0.47***	0.58***	0.33***	0.44***	0.36***	0.46***	0.35***	0.46***
Rio de Janeiro	0.35***	0.41***	0.35***	0.41***	0.33***	0.37***	0.34***	0.41***	0.36***	0.43***	0.29***	0.33***
Cape Town	0.27***	0.30***	0.25***	0.33***	0.31***	0.33***	0.26***	0.31***	0.24***	0.26***	0.26***	0.28***

Note: Statistical significance is indicated by asterisks (* $p < 0.05$, ** $p < 0.01$, *** $p < 0.001$).

Supplementary Table 6: Average ρ values with varying library sizes L reflecting the causal influence between urban systems and traffic dynamics on work days across 30 cities. Results are presented for library sizes of 100 and 500, except for Manila, where the maximum available library size is below 500 and the ρ value from the largest available size is used. Statistical significance was evaluated using a one-sample Student's t-test on the distribution of ρ values across all BVCells at each library size. All statistical tests were two-sided. Exact p-values for all statistical tests are provided in the source data files.

City	Urban system → Traffic dynamics						Traffic dynamics → Urban system					
	Structure		Form		Function		Structure		Form		Function	
	100	500	100	500	100	500	100	500	100	500	100	500
London	0.35***	0.40***	0.38***	0.46***	0.36***	0.42***	0.33***	0.41***	0.33***	0.41***	0.33***	0.41***
Paris	0.31***	0.41***	0.33***	0.45***	0.36***	0.47***	0.31***	0.39***	0.30***	0.40***	0.31***	0.40***
Zurich	0.28***	0.12***	0.26***	0.08***	0.32***	0.13***	0.19***	0.17***	0.17***	0.17***	0.20***	0.16***
Rome	0.30***	0.37***	0.28***	0.34***	0.32***	0.39***	0.25***	0.29***	0.26***	0.31***	0.26***	0.28***
Vienna	0.21***	0.28***	0.24***	0.32***	0.23***	0.31***	0.24***	0.26***	0.24***	0.28***	0.25***	0.29***
Berlin	0.26***	0.30***	0.27***	0.36***	0.28***	0.33***	0.30***	0.32***	0.27***	0.29***	0.31***	0.31***
Stockholm	0.13***	0.10***	0.11***	0.10***	0.15***	0.13***	0.16***	0.10***	0.16***	0.07***	0.17***	0.10***
Helsinki	0.34***	0.40***	0.33***	0.40***	0.36***	0.43***	0.27***	0.38***	0.29***	0.40***	0.29***	0.41***
St. Petersburg	0.28***	0.35***	0.34***	0.41***	0.32***	0.39***	0.28***	0.30***	0.26***	0.30***	0.28***	0.31***
Moscow	0.26***	0.26***	0.26***	0.27***	0.32***	0.31***	0.26***	0.23***	0.22***	0.20***	0.27***	0.25***
Riyadh	0.30***	0.36***	0.26***	0.37***	0.40***	0.45***	0.26***	0.30***	0.19***	0.25***	0.28***	0.32***
Dubai	0.35***	0.36***	0.31***	0.35***	0.35***	0.35***	0.32***	0.34***	0.27***	0.30***	0.32***	0.34***
New Delhi	0.44***	0.58***	0.46***	0.63***	0.52***	0.66***	0.37***	0.51***	0.34***	0.46***	0.39***	0.53***
Mumbai	0.34***	0.42***	0.31***	0.38***	0.39***	0.45***	0.33***	0.41***	0.31***	0.43***	0.34***	0.38***
Bangkok	0.33***	0.37***	0.26***	0.29***	0.37***	0.39***	0.30***	0.33***	0.25***	0.26***	0.29***	0.32***
Hong Kong	0.26***	0.30***	0.30***	0.35***	0.30***	0.36***	0.28***	0.36***	0.26***	0.37***	0.29***	0.38***
Manila	0.30***	0.26***	0.33***	0.29***	0.32***	0.26***	0.32***	0.31***	0.30***	0.27***	0.32***	0.33***
Kuala Lumpur	0.30***	0.34***	0.30***	0.33***	0.35***	0.38***	0.23***	0.25***	0.22***	0.22***	0.25***	0.25***
Singapore	0.37***	0.43***	0.40***	0.47***	0.40***	0.45***	0.29***	0.36***	0.29***	0.34***	0.30***	0.37***
Jakarta	0.43***	0.48***	0.41***	0.47***	0.39***	0.39***	0.36***	0.42***	0.34***	0.42***	0.32***	0.39***
Sydney	0.14***	0.10***	0.15***	0.09***	0.17***	0.10***	0.18***	0.13***	0.19***	0.15***	0.19***	0.16***
Christchurch	0.36***	0.40***	0.39***	0.43***	0.49***	0.51***	0.39***	0.48***	0.39***	0.47***	0.41***	0.48***
Los Angeles	0.23***	0.16***	0.24***	0.21***	0.25***	0.18***	0.21***	0.18***	0.19***	0.18***	0.22***	0.20***
Chicago	0.24***	0.26***	0.25***	0.27***	0.30***	0.29***	0.22***	0.24***	0.19***	0.24***	0.23***	0.26***
Toronto	0.23***	0.27***	0.26***	0.28***	0.30***	0.35***	0.24***	0.28***	0.21***	0.27***	0.26***	0.32***
Mexico City	0.35***	0.40***	0.32***	0.38***	0.32***	0.43***	0.29***	0.34***	0.28***	0.32***	0.17***	0.21***
Lima	0.26***	0.29***	0.28***	0.27***	0.32***	0.30***	0.26***	0.27***	0.24***	0.28***	0.26***	0.27***
Buenos Aires	0.33***	0.38***	0.35***	0.43***	0.39***	0.45***	0.29***	0.39***	0.30***	0.40***	0.31***	0.41***
Rio de Janeiro	0.30***	0.28***	0.30***	0.32***	0.35***	0.25***	0.28***	0.29***	0.28***	0.29***	0.27***	0.29***
Cape Town	0.18***	0.15***	0.20***	0.13***	0.20***	0.13***	0.18***	0.13***	0.18***	0.11***	0.17***	0.15***

Note: Statistical significance is indicated by asterisks (* $p < 0.05$, ** $p < 0.01$, *** $p < 0.001$).

Supplementary Table 7: Statistical assessment of the causal asymmetry ($\Delta\rho$) between urban systems and traffic dynamics across 30 cities. The table reports the mean difference in cross-mapping skill at the largest library size, defined as $\Delta\rho = \rho_{\text{urban systems}\rightarrow\text{traffic dynamics}} - \rho_{\text{traffic dynamics}\rightarrow\text{urban systems}}$. A positive value indicates that urban systems exert a stronger causal influence on traffic dynamics, while a negative value implies the reverse. Statistical significance was evaluated using a paired Student's t-test comparing the distributions of causal strengths in both directions across all BVCells for each city. All statistical tests were two-sided. Exact p-values for all statistical tests are provided in the source data files.

City	Rest days			Work days		
	Structure $\Delta\rho$	Form $\Delta\rho$	Function $\Delta\rho$	Structure $\Delta\rho$	Form $\Delta\rho$	Function $\Delta\rho$
London	0.011***	0.071***	0.054***	-0.002	0.052***	0.013***
Paris	0.013***	0.046***	0.076***	0.016***	0.048***	0.063***
Zurich	-0.011	0.047***	0.059***	-0.045***	-0.096***	-0.030***
Rome	0.052***	0.077***	0.090***	0.084***	0.031***	0.110***
Vienna	0.004	-0.004	0.014***	0.011**	0.040***	0.024***
Berlin	0.010***	0.028***	0.042***	-0.019***	0.063***	0.021***
Stockholm	0.039***	0.049***	0.033***	0.004	0.028***	0.030***
Helsinki	0.019***	0.031***	0.018***	0.016***	0.005*	0.018***
St. Petersburg	0.048***	0.079***	0.086***	0.054***	0.109***	0.079***
Moscow	0.033***	0.109***	0.065***	0.030***	0.076***	0.058***
Riyadh	0.063***	0.132***	0.101***	0.062***	0.119***	0.134***
Dubai	0.019***	0.044***	0.043***	0.021***	0.043***	0.015***
New Delhi	0.054***	0.122***	0.120***	0.073***	0.165***	0.133***
Mumbai	0.015***	-0.023***	0.097***	0.005*	-0.053***	0.072***
Bangkok	0.055***	0.024***	0.067***	0.043***	0.033***	0.068***
Hong Kong	-0.010***	0.018***	0.000	-0.061***	-0.011***	-0.023***
Manila	0.030***	-0.072***	0.036***	-0.048***	0.021***	-0.068***
Kuala Lumpur	0.063***	0.055***	0.132***	0.093***	0.105***	0.123***
Singapore	0.043***	0.090***	0.063***	0.071***	0.122***	0.087***
Jakarta	0.056***	0.030***	-0.001	0.054***	0.046***	-0.003
Sydney	0.157***	0.139***	0.186***	-0.037***	-0.059***	-0.054***
Christchurch	-0.054***	0.020***	-0.005	-0.081***	-0.037***	0.028***
Los Angeles	0.005***	0.042***	-0.014***	-0.021***	0.026***	-0.019***
Chicago	0.023***	0.078***	0.087***	0.020***	0.040***	0.031***
Toronto	0.015***	0.041***	0.025***	-0.015***	0.016***	0.030***
Mexico City	-0.005**	0.009***	0.023***	0.060***	0.056***	0.225***
Lima	0.001	-0.004*	0.015***	0.019***	-0.008***	0.029***
Buenos Aires	0.049***	0.093***	0.116***	-0.007**	0.034***	0.036***
Rio de Janeiro	-0.002	-0.017***	0.044***	-0.006**	0.027***	-0.035***
Cape Town	-0.013***	0.069***	0.055***	0.019***	0.023***	-0.025***

Note: Statistical significance is indicated by asterisks (* $p < 0.05$, ** $p < 0.01$, *** $p < 0.001$). Values without asterisks indicate no statistical significance ($p \geq 0.05$).

Supplementary Table 8: Spatial and temporal details of the HERE traffic dataset for 30 global cities. The segment count denotes the number of HERE road segments within each city's boundary. The time period refers to the one-month windows from which 5-minute traffic data were collected: 1 Jun - 30 Jun 2024 or 21 Sept - 20 Oct 2024. The time-coverage ratio is the proportion of all 5-minute slots in the window for which valid traffic data are available. The spatial-coverage ratio is computed on a 1 km $\times$ 1 km grid and measures spatial representativeness relative to the OSM road network: for each grid containing OSM roads for motor-vehicle traffic, we report the percentage that also contains HERE road segments. This information is visualized in the spatial heatmap, where grids with only OSM roads are shown in grey and those with both HERE and OSM roads in blue. City boundaries follow the latest GADM administrative units, except for Singapore, whose official boundary is sourced from Singapore's Urban Redevelopment Authority due to the outdated GADM.

City	London	Paris	Zurich	Rome	Vienna
Segment count	4,045	2,802	1,241	3,422	3,150
Time period	01 Jun-30 Jun	01 Jun-30 Jun	01 Jun-30 Jun	01 Jun-30 Jun	21 Sept-20 Oct
Time coverage	98.58%	98.63%	98.58%	98.61%	99.87%
Spatial coverage	80.15%	94.57%	85.58%	74.85%	82.56%
Spatial heatmap	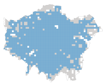	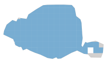	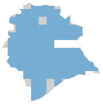	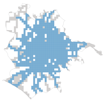	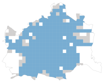

City	Berlin	Stockholm	Helsinki	St. Petersburg	Moscow
Segment count	3,504	2,001	4,876	7,426	15,463
Time period	21 Sept-20 Oct	21 Sept-20 Oct	21 Sept-20 Oct	21 Sept-20 Oct	21 Sept-20 Oct
Time coverage	99.86%	99.85%	99.85%	99.87%	99.87%
Spatial coverage	86.38%	81.19%	85.20%	82.43%	90.10%
Spatial heatmap	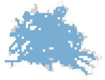	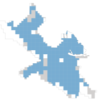	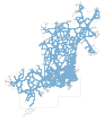	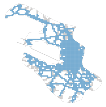	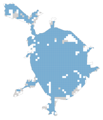

City	Riyadh	Dubai	New Delhi	Mumbai	Bangkok
Segment count	7,726	4,749	9,121	2,054	11,566
Time period	21 Sept-20 Oct	21 Sept-20 Oct	21 Sept-20 Oct	01 Jun-30 Jun	01 Jun-30 Jun
Time coverage	99.86%	99.86%	99.87%	98.55%	98.58%
Spatial coverage	50.58%	74.16%	82.91%	90.70%	91.47%
Spatial heatmap	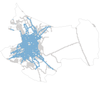	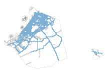	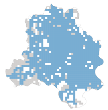	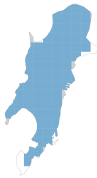	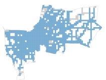

City	Hong Kong	Manila	Kuala Lumpur	Singapore	Jakarta
Segment count	3,661	944	3,224	5,512	8,302
Time period	01 Jun-30 Jun	01 Jun-30 Jun	21 Sept-20 Oct	01 Jun-30 Jun	01 Jun-30 Jun
Time coverage	98.51%	98.54%	99.86%	98.51%	98.53%
Spatial coverage	75.68%	89.80%	86.85%	90.28%	93.15%
Spatial heatmap	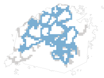	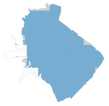	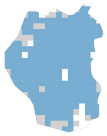	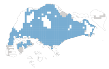	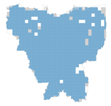

City	Sydney	Christchurch	Los Angeles	Chicago	Toronto
Segment count	991	2,427	18,788	12,298	3,392
Time period	01 Jun-30 Jun	01 Jun-30 Jun	21 Sept-20 Oct	21 Sept-20 Oct	01 Jun-30 Jun
Time coverage	98.58%	98.54%	99.85%	99.87%	98.54%
Spatial coverage	94.74%	91.09%	83.96%	90.41%	93.54%
Spatial heatmap	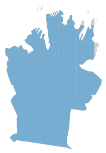	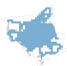	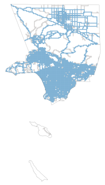	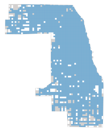	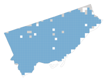

City	Mexico City	Lima	Buenos Aires	Rio de Janeiro	Cape Town
Segment count	2,807	9,683	3,157	6,235	3,467
Time period	01 Jun-30 Jun	01 Jun-30 Jun	01 Jun-30 Jun	01 Jun-30 Jun	21 Sept-20 Oct
Time coverage	98.41%	98.40%	98.58%	98.59%	99.86%
Spatial coverage	63.17%	75.72%	94.81%	90.87%	81.57%
Spatial heatmap	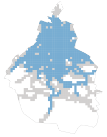	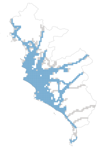	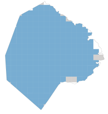	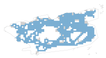	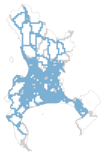

Supplementary Table 9: Descriptions of features characterizing urban structure, form, and function.

Abbreviation	Full Name	Definition
NODE_CT	Node Count	The number of nodes in the road network.
NODE_DENS	Node Density	The number of nodes per unit area.
CRS_CT	Crossing Count	The number of road intersections.
K_AVG	Average Node Degree	The average degree of nodes in the road network.
EDGE_LEN	Total Length	The total length of road segments.
RD_DENS	Road Density	The total road length per unit area.
NET_INT	Network Intensity	The total length of road segments normalized by the cell perimeter.
BTW_CEN	Average Betweenness Centrality	The average of the fraction of shortest paths that pass through each node.
CLS_CEN	Average Closeness Centrality	The average reciprocal of the sum of the shortest path distances from a node to all other nodes.
DEG_CEN	Average Degree Centrality	The average normalized degree of nodes within the cell.
TA	Total Area	The total area of land-use patches.
NP	Number of Patches	The total number of land-use patches.
PD	Patch Density	The number of land-use patches per unit area.
TE	Total Edge	The total length of all patch edges.
ED	Edge Density	The total length of all patch edges per unit area.
LSI	Landscape Shape Index	The measure of landscape shape complexity.
PARA	Perimeter-Area Ratio	The average perimeter-area ratio across all patches within the cell.
SHAPE	Shape Index	The average shape index across all patches, quantifying how much patch shapes deviate from compact forms.
FRAC	Fractal Dimension	The average fractal dimension of all patches, describing how perimeter complexity scales with patch size.
CONTAG	Contagion	The degree to which land-use patches are clumped or dispersed.
AI	Aggregation Index	The measure indicating how closely land-use patches are clustered together.
COHES	Cohesion	The physical connectedness of land-use patches within the cell.
SPLIT	Splitting Index	The fragmentation metric reflecting the degree of landscape subdivision.
PR	Patch Richness	The total number of distinct patch types.
SHDI	Shannon's Diversity Index	The diversity of area distribution among patch types.
SHEI	Shannon's Evenness Index	The evenness of area distribution among patch types.
TRNS	Transportation Area	The percentage of land designated for transport infrastructure.
COMM	Commercial and Business Facilities Area	The percentage of land used for commerce, finance, hospitality, and retail.
IND	Industrial Area	The percentage of land used for industrial production and extraction.

Abbreviation	Full Name	Definition
RES	Residential Area	The percentage of land designated for housing and living quarters.
FRST	Farmland and Forest Area	The percentage of natural, semi-natural, and productive rural land.
EDU	Education and Science Area	The percentage of land dedicated to educational and research institutions.
SPRT	Sports Area	The percentage of land used for physical sports and athletic activities.
PARK	Park Area	The percentage of public green spaces and manicured vegetation.
RECR	Recreation Area	The percentage of land used for leisure, tourism, and entertainment.
WATR	Water Area	The percentage of land covered by water bodies.
HLTH	Health Care Area	The percentage of land used for medical and care services.
CULT	Cultural Facilities Area	The percentage of land used for cultural arts and public gatherings.
ADMIN	Administration Area	The percentage of land used for government and public administration.
